# Cost-Effectiveness of Screening and Treating Foreign-Born Students for Tuberculosis before Entering the United States

**DOI:** 10.1371/journal.pone.0124116

**Published:** 2015-04-29

**Authors:** La’Marcus T. Wingate, Margaret S. Coleman, Drew L. Posey, Weigong Zhou, Christine K. Olson, Brian Maskery, Martin S. Cetron, John A. Painter

**Affiliations:** Division of Global Migration and Quarantine; Centers for Disease Control and Prevention; Atlanta, Georgia, United States of America; McGill University, CANADA

## Abstract

**Introduction:**

The Centers for Disease Control and Prevention is considering implementation of overseas medical screening of student-visa applicants to reduce the numbers of active tuberculosis cases entering the United States.

**Objective:**

To evaluate the costs, cases averted, and cost-effectiveness of screening for, and treating, tuberculosis in United States-bound students from countries with varying tuberculosis prevalence.

**Methods:**

Costs and benefits were evaluated from two perspectives, combined and United States only. The combined perspective totaled overseas and United States costs and benefits from a societal perspective. The United States only perspective was a domestic measure of costs and benefits. A decision tree was developed to determine the cost-effectiveness of tuberculosis screening and treatment from the combined perspective.

**Results:**

From the United States only perspective, overseas screening programs of Chinese and Indian students would prevent the importation of 157 tuberculosis cases annually, and result in $2.7 million in savings. From the combined perspective, screening programs for Chinese students would cost more than $2.8 million annually and screening programs for Indian students nearly $440,000 annually. From the combined perspective, the incremental cost for each tuberculosis case averted by screening Chinese and Indian students was $22,187 and $15,063, respectively. Implementing screening programs for German students would prevent no cases in most years, and would result in increased costs both overseas and in the United States. The domestic costs would occur because public health departments would need to follow up on students identified overseas as having an elevated risk of tuberculosis.

**Conclusions:**

Tuberculosis screening and treatment programs for students seeking long term visas to attend United States schools would reduce the number of tuberculosis cases imported. Implementing screening in high-incidence countries could save the United States millions of dollars annually; however there would be increased costs incurred overseas for students and their families.

## Introduction

Tuberculosis (TB) is a serious disease with long-term health and economic consequences. United States TB incidence rates are heavily driven by foreign-born persons, who account for over 60% of new cases [1]. The TB incidence rate among all foreign-born persons in the United States is 15.8 cases per 100,000 [1], as compared with 3.2 in the total domestic population that includes the foreign-born. The highest incidence rates among the foreign-born occur within the first year of United States arrival. Data from 2001 through 2006 indicate that disease rates approach 120 cases per 100,000 in foreign-born persons during their first year in the United States [2].

The Centers for Disease Control and Prevention (CDC)’s Division of Global Migration and Quarantine (DGMQ) is responsible for the regulations and policies that are used to prevent the introduction, transmission, and interstate spread of communicable diseases into the United States [3]. Under current regulations, approximately 500,000 immigrants and refugees annually seek permanent residence, and it is mandatory that those seeking permanent residence receive overseas medical examinations that include TB testing as part of their visa application process to seek permission to emigrate [4–7]. Applicants diagnosed overseas with active TB as a result of testing are treated before being allowed to enter the United States. CDC data indicate that over 1,100 cases of TB were detected in 2012, with those applicants stopped from entering the United States until their TB was treated [8].

There are a total of 300 temporary entrants, known as nonimmigrant visa-holders (NIVs) for every person seeking permanent residence[9]; and annually there are approximately 160 million temporary new admissions to the United States for work, school, business, or leisure. NIV applicants are not required to undergo the same medical screening with TB testing as persons seeking permanent residence, such as immigrants and refugees. However, many NIVs hail from countries that have high rates of TB disease and they represent a reservoir of TB cases that regularly enters the United States [10].

NIVs can be divided into two categories: short-term visitors (e.g., tourists and business); and long-term visitors (e.g., students, temporary workers and exchange visitors) who reside in the United States for months or years [10,11]. It is impractical to test short-term NIVs for TB before domestic arrival because of the large volume (160 million annual admissions), and the yield in cases detected would likely be very low [9,10]. However, CDC hypothesizes that testing long-term NIVs, (defined as persons residing in the United States for ≥ 6 months,) would probably prevent importation of enough active TB cases to make a strong and positive impact on domestic public health [10]. Implementation of any new medical screening program overseas to include TB testing would require a change in domestic federal regulations governing the medical tests administered overseas because current laws only cover those seeking permanent entry to the United States. Before proposing additional medical screening regulations, CDC is interested in studying the costs and effectiveness of administering TB screening to a new, much larger population. Therefore, we modeled the epidemiologic benefits and cost-effectiveness of implementing an overseas medical program where student-visa applicants seeking temporary entry to the United States are screened for TB. Hereafter, student-visa applicants are referred to as students.

## Methods

Our model evaluates costs, benefits, and effectiveness of overseas vs. no overseas TB screening of foreign students while still in their native countries. The public health outcome measure of preventing importation of active TB cases into the United States is estimated from the domestic (United States only) perspective. The ‘combined’ measure includes both domestic and overseas costs. The primary measure was an incremental cost effectiveness ratio (ICER) that totals domestic and overseas costs as compared with an outcome of tuberculosis cases prevented from being imported into the United States (the combined perspective). The ‘combined’ analysis can be said to have been conducted from a partial societal perspective insomuch as both overseas and domestic costs accruing to the health system, patients, and universities are considered in the analysis. However, to be a complete societal perspective we would have had to include the impact of the program on the entire domestic population and that was beyond the scope of this study. Our methods section starts with model components used in both domestic and overseas calculations; and, then we explain the specifics of overseas and domestic components in sequence. Further methods details are available in the S1 Appendix.

### Student population

For our hypothetical study cohort, we focused on student populations from three countries with wide ranges of TB rates: China, India and Germany. China and India were the top two countries of origin for all foreign-born students, while Germany sent the most students from European countries [12]. Nearly 30% of all foreign-born students were newly enrolled [13], and we assumed that newly enrolled students would also be in their first year in the United States. Of 194,029 Chinese, 100,270 Indian, and 9,347 German students, our hypothetical newly enrolled student cohort (~30% of total students) contained: 58,015 Chinese, 29,981 Indians, and 2,795 Germans [12].

### Cost-effectiveness model

We constructed a decision tree using TreeAge Pro Software 2012 (Williamstown, MA) and cost components were estimated in Microsoft Excel. We compared how the total costs and outcome of numbers of TB cases imported into the United States differed in two models: ‘overseas TB screening’ and ‘no overseas TB screening’ (where both scenarios included some domestic TB treatment). The cost-effectiveness model resulted in an ICER that compared changes in costs and the reduction (avoidance) of numbers of TB cases imported that resulted from no overseas screening as compared to overseas screening by country of origin. The ICER formula incorporated the costs of screening and treatment and the outcome of avoided TB cases: [(cost of overseas screening)–(cost of no overseas screening)]/ [(TB cases diagnosed in United States with no overseas screening)–(TB cases diagnosed in United States with overseas screening)] (This formula is for TB disease/cost avoided; see S1 Appendix for an example of an ICER formula that would be calculated for benefits expressed as a gain). Our model used a societal (‘combined’) perspective for the number of TB cases that occurred for the time-period the student underwent overseas screening until the end of their first year in the United States. No discounting was applied because all values were estimated only for the first year.

### Cost components

We calculated the following both overseas and domestically: student opportunity costs, hospitalizations, direct medical costs for TB screening and associated treatment (panel physician overseas, Public Health Departments (PHDs) in the United States), and totals for domestic and overseas estimates.

The costs components specific to the United States were: DGMQ expenses to coordinate TB follow-up activities, lost tuition for hospitalized students, and burden to insurers for hospitalizations.

Where there was no screening there were no overseas program costs; the only costs were domestic TB treatment.

All costs and charges were expressed in 2011 United States dollars.

### Overseas: processes and costs

#### Screening process

Students would be screened and treated, where necessary, for active TB by ‘panel physicians’ according to the most recent Directly Observed Therapy Technical Instructions for testing and treating TB, or Culture and Directly Observed Therapy Tuberculosis Technical Instructions (CDOT TB TI) [7] (see S1 Appendix). After completing overseas medical screening and other entry requirements, student-applicants with no indication of TB received no TB medical classification on their entry visa. Otherwise, they received either a Class A or Class B-1 medical classification on their visa as a result of the CDOT TBTI medical tests [4,7,8] (see S1 Appendix). Students diagnosed with active TB disease (Class A visa medical classifications) must go through CDC-approved medical treatment for TB and be retested for disease before they would be allowed to re-apply to enter the United States.

Our analysis focused on overseas screening for active TB and did not evaluate the role of latent TB infection (LTBI) because current regulations only require medical screening for TB. Medically there is certainly a direct, epidemiologic/medical connection between TB and LTBI; but LTBI was not included in our study because there is no current legal mandate or regulation to screen for LTBI in any group seeking entry to the United States (e.g., immigrants, refugees, or NIVs).

#### Data

Our primary source of disease outcome data was TB Indicator Data documenting information for immigrant and refugee populations by country. TB Indicator Data included country-specific numbers of populations that underwent TB screening. China and India had data available in the 2012 TB Indicator Data, and for these two countries we assumed students would have the same TB screening outcome rates as the general population (e.g., for no disease, active disease, or suspected/previous disease). This was a safe assumption because the vast majority of the Chinese and Indian screened populations are immigrants who would need to also prove economic viability for their full visa application process. We assumed families that were able to send children abroad for study would be similar to immigrant populations. Unfortunately, Germany was not included in the TB Indicator Data [7]; however, we assumed data for Canada and France, where TB disease rates are low [14], would adequately represent German students in the model. Therefore, we averaged the TB screening and disease outcomes data from Canadian and French applicants for German students.

With regards to the proportion of students that completed treatment following a finding of active TB disease, we used only Chinese TB Indicator Data because CDC professionals deemed the Chinese treatment completion data to be the most accurate. We estimated that 54% of all students diagnosed with TB also completed panel physician treatment and included the costs of panel physician treatment in the model. We did not include treatment costs at locations other than panel physicians’ offices, or costs of no treatment. Students seeking treatment outside the panel physician system or not getting treated would be ineligible to enter the United States.

#### Costs associated with screening and treatment

For overseas TB screening and treatment, panel physician charges equated to students’ costs to receive TB disease testing as part of the same standard, comprehensive medical examination currently required of immigrants and refugees. Therefore, we needed to estimate the portion of the panel physicians’ medical examination charges specific to TB screening and treatment by using immigrant medical examination charges (Table 1). In India and Germany, the panel physician examination charges were separated into three components: syphilis, chest radiograph and consult, and general physical examination. During the physical examination, patients were evaluated for several conditions, including signs and symptoms of TB disease, so we pro-rated the examination charge to 30% for the TB-specific protocols. Therefore, for the TB-specific portion of the entire exam, we included the entire fee for the chest radiograph and radiology consult and apportioned 30% of the physical examination charge. Using this methodology, the TB-specific screening charges were 36% of the total exam charges in India and 40% of the total exam charges in Germany. In China, there was only a flat-rate panel physician medical examination charge. For China, we apportioned 38% of the total examination charges in order to determine the TB-specific charge; setting the China cost at the midpoint of the TB-specific examination charges for India and Germany. Panel physicians also supplied data regarding charges for active TB tests and treatment for applicants found to have active TB disease during their overseas examinations. Hospitalization and medication costs were estimated from the literature and WHO Choice data [15–21].

**Table 1:** Overseas Parameters in Cost-Effectiveness Model Comparing Programs for Screening and Treating TB in Foreign-Born Student Visa-Applicants.

Parameter	India	China	Germany	Source
Number in hypothetical cohort	29,981	58,015	2,795	[12,13]
% with suspected TB^A^	2.7%	3.8%	0.6%^B^	^TB Indicator Data^
% active disease in suspected TB	3.6%	5.8%	0%^B^	^TB Indicator Data^
Cases diagnosed^C^	29	128	0	^Calculated^
% initiating and completing treatment^D^	54%	54%	NA	^Panel Physicians^
Cost of initial TB screening^E^	$15.95	$54.94	$72.93	^Panel PhysiciansPhysicians^
Cost for three sputum smears and cultures^A^	$113.57	$153.79	$155.88	^Panel Physicians^
Student opportunity cost for initial TB screening^E^	$9.61	$22.36	$102.17	[24]
Student opportunity cost for three sputum smears and cultures^A^	$28.55	$66.60	$302.05	[24]
Cost for Panel Physician monitoring and supervision^F^	$717.70	$611.74	NA	^Panel Physicians^
Cost for medications^F^	$48.20	$45.31	NA	[17–19]
Average hospitalization cost per case^F^	$39.80	$125.28	NA	[15–17,20,21]
Opportunity cost for directly observed therapy and monitoring at panel physicians	$125.45	$288.93	NA	[24]
Opportunity cost for disease impairment	$159.00	$365.92	NA	[24,25]
Opportunity cost for reapplying to college after treatment	$1.67	$3.83	NA	[24]

NA = Not applicable because no cases were detected in German students; TB = Tuberculosis

A-Suspected TB includes those with abnormal chest radiograph, signs and symptoms of TB, or known HIV infection, and these persons undergo three sputum smears and cultures;

B-Reflects the proportion seen in the low incidence countries of France and Canada;

C-Calculated by multiplying number in cohort times % suspected TB times % active disease among those with suspected TB;

D-Based upon data submitted by panel physicians in China;

E-Initial TB screening consists of chest radiograph and part of physical examination;

F-costs not available for German students because the modeled results indicate that no cases would be detected in German students

#### Opportunity costs

The cost of each activity is the activity not engaged in, or the opportunity costs [22,23]. Student hourly time value was estimated by dividing the country-specific Gross Domestic Product (GDP) per capita adjusted for purchasing power parity (PPP) by annual hours [24]. Chinese and Indian students receiving TB treatment were assigned an opportunity cost for numbers of disease impairment days based on published data for India [25]. No German students were diagnosed with TB. We assumed all students who started and completed CDC-approved treatment would reapply for school and successfully obtain visas, so we added four hours of opportunity costs for students’ reapplication.

### Domestic: processes and costs

#### Follow-up processes for Class B-1 medical visa designations and domestic data

CDC recommends, but does not mandate that anyone with a Class B-1 medical visa designation report for medical follow-up for further TB testing. In order to estimate the number of students with Class B-1 medical visa designations that would follow-up, we estimated how many immigrants presented to follow-up, and what type of follow-up TB testing they received. We then assumed that students presented for follow-up at the same rate as immigrants and received the same tests.

In order to make estimates regarding immigrants, we used raw data from the Electronic Disease Notification system (EDN) along with publications that analyzed EDN data. EDN notifies local public health officials of arriving immigrants and refugees with a TB-related medical visa designation and allows the same local officials to report the outcomes of follow-up examinations [26]. Our model used a midpoint result of 79% from the literature [4] applied to students to estimate how many would report for domestic follow-up exams (Table 2).

**Table 2:** U.S.-Specific Parameters in Cost-Effectiveness Model Comparing Programs forTreating TB in Foreign-Born Student-Visa Applicants.

Parameter Value in Class B-1 Students^A^	Base Value	Source
% Class B-1 students presenting to follow-up^A^	78.6%	[4]
% Indian Class B-1 students diagnosed with TB after U.S. arrival	2.30%	^EDN Data^
% Chinese Class B-1 students diagnosed with TB after U.S. arrival	1.67%	^EDN Data^
% German Class B-1 students diagnosed with TB after U.S. arrival	0%	^EDN Data^
% Class B-1 students with chest radiograph repeated at follow-up	91.3%	^EDN Data^
% Class B-1 students receiving sputum smears and cultures after initial chest radiograph	69.7%	^EDN Data^
Hospitalization rate in students with TB detected during follow-up	30.6%	[47,49]
Hospitalization rate in students with TB passively	49.0%	[47,49]

EDN = Electronic Disease Notification System; TB = Tuberculosis; U.S. = United States

A-Class B-1 indicates those who have an abnormal chest radiograph, signs and symptoms of TB, or known HIV infection during overseas screening and are encouraged to follow up at U.S. public health department

EDN also contains information on the proportion of immigrants with a Class B-1 medical designation on their visa who ultimately get domestically diagnosed with active TB [26]. We used the EDN data for Chinese and Indian immigrants (unpublished data held by CDC) to estimate the percentage of those students who would be domestically diagnosed with active TB. None of the German immigrants with a Class B-1 medical designation on their visa were diagnosed with TB, so we assumed that none of the German students would be diagnosed with TB either.

#### Domestic costs

PHD costs were estimated using components for physician and nurse time to evaluate patients [27–30] and diagnostic test prices [31–33] (Table 3). Medical staff salaries were adjusted for benefits by adding 27% to cash wages [34]; and then added 30% of the sum of salary and benefits and diagnostic test prices for non-specified overhead. Finally, we assumed 5% of total PHD costs would be an adequate estimate of DGMQ resources.

**Table 3:** Components for U.S. Costs Associated with Follow-Up of Class B-1 Students and Treatment of TB Cases.

Component	Item	Cost	Source
Initial visit in Class B-1 students^A^ ^,^ ^B^	Chest radiograph	$30.24	[33]
	1 hour nurse time	$45.77	[27,34]
	30 minutes physician time	$62.65	[27,34]
	2 hours student time	$10.98	[24]
	Student fuel cost	$2.91	[36–38 ]
Second visit in Class B-1 students^A^ ^,^ ^B^	Medical tests^C^	$105.88	[32]
	1 hour nurse time	$45.77	[27,34]
	30 minutes physician time	$62.65	[27,34]
	2 hours student time	$10.98	[24]
	Student fuel cost	$2.91	[36–38 ]
Opportunity cost due to disease impairment	12.5 days of disease related impairment	$1,647.00	[24,44]
Diagnosis^B^	Medical tests^D^	$175.92	[32,33]
	2 hours nurse time	$91.54	[27,29,34]
	1 hour physician time	$125.30	[27,29,34]
	4 hours student time	$21.96	[25]
	Student fuel cost for two trips	$5.82	[36–38 ]
Contact tracing^B^	Health worker time for identification of 10 contacts	$961.50	[39,40]
DOT when PHD delivers medicine to student for 130 days (60% of cases) ^B^	45 minutes outreach worker time	$20.45	[27,34]
	Outreach worker fuel cost	$2.91	[36–38]
	8 minutes patient time for medicine	$0.73	[24,28,29]
	Daily cost	$24.09	
DOT when patient travels to PHD for 130 days (40% of cases)^B^	8 minutes outreach worker time	$3.63	[27–29,34]
	1 hour student time	$5.49	[24]
	Student fuel cost	$2.91	[36–38 ]
	Daily cost	$12.03	
Medications^B^	6 months of therapy	$431.97	[41,42]
Monthly follow-up visits^B^	Medical tests^E^	$109.30	[32,33]
	30 minutes of nursing time at each of 5 visits	$114.43	[27,28,34]
	1.5 hours of student time at each of 5 visits	$41.18	[24,28]
	Student fuel costs for 5 visits	$14.55	[36–38]
Hospitalization	Average cost for TB-related inpatient treatment	$19,481.00	[45]
	Opportunity cost for 13 days while hospitalized	$1,713.58	(25,44)
Costs of forfeited tuition	Students hospitalized with TB^F^	$7,418.78	[49]

DOT = Directly Observed Therapy; PHD = Public Health Department; TB = Tuberculosis; U.S. = United States

A-Class B-1 indicates those who have an abnormal chest radiograph, signs and symptoms of TB, or known HIV infection during overseas screening and are encouraged to follow up at U.S. PHD;

B-In final analysis, 30% added to all PHD incurred costs to account for overhead;

C-Includes 3 sputum smears, 3 cultures, a complete blood count, serum chemistry test, and baseline liver enzymes;

D-Includes 3 sputum smears, 3 cultures, a complete blood count, serum chemistry test, drug sensitivity test, chest radiograph and baseline liver enzymes;

E-Includes 2 chest radiographs, 2 cultures, and monthly liver function tests for 25% of patients;

F-Assumes hospitalized students forfeit 70% of tuition

For the 79% of students who reported for follow-up, we estimated an opportunity cost by dividing the 2011 United States per capita GDP adjusted for PPP by 8,760 and obtained an hourly value of $5.49 [24], plus travel expenses to reflect follow-up visit time used and, where medically indicated, TB treatment [35–38].

TB outpatient costs at PHDs included: physician and nurse time [27–30], diagnostic tests [31–33], contact tracing [39,40], monthly follow-up nursing visits [32,33], and six months of medications [41,42], assuming all patients were treatable with first-line medications [43]. All students domestically diagnosed with TB were assigned 12.5 days of opportunity cost for disease impairment [44].

All patients received DOT treatment (Table 3); if PHDs traveled to patients, costs were included for outreach worker travel and medication administration time [27–30], transportation [35–38], and student opportunity costs to take medication [27–30]; when students traveled to PHDs, they incurred opportunity costs for traveling and medication administration [27–30] and travel expenses [35–38], while the PHD incurred medication administration costs [27–30].

The average length and cost of a TB hospital stay was estimated using published data [45]. We assumed a 49% hospitalization rate for TB cases detected passively, i.e., when a patient seeks medical attention for symptoms instead of being tested for disease [46]. TB cases detected through domestic follow-up testing (active identification) were assumed to have lower hospitalization rates because patients would be treated earlier in the disease progression [47,48]. As most students are required to have insurance, we assumed 80% of hospitalization costs would be borne by insurance companies. In addition we assumed that hospitalized students would withdraw from school for one semester, forfeiting 70% of their tuition and fees [49], though we acknowledge different schools had different policies [50–55].

### Sensitivity analysis of cost-effectiveness model

Our sensitivity analysis evaluated the effect of changing one parameter at a time on the ICER results for China and India by choosing parameters with high degrees of uncertainty or those that would impact the results in a substantive manner:

- Plus or minus 50% of students suspected of having TB;
- Varied the proportion of Class B-1 students domestically diagnosed with TB from 0.3% to 3%;
- Varied tuition forfeited by domestically hospitalized students from 0% to 100%;
- Added a higher rate of tuition to account for graduate students [56];
- Plus or minus 50% of the United States based hospitalization costs.
  We also performed a sensitivity analysis where we excluded all opportunity cost.

### Institutional review board approval

The study was submitted to the CDC institutional review board and deemed exempt from review.

## Results

The impact of implementing an overseas TB screening program in China and India would be substantial on the reduction in domestic imported TB cases, while the impact of the same program in Germany would be negligible (Table 4). Without screening, 210 students with TB cases annually would enter the United States from China and India and that implementing overseas screening would reduce the number of TB cases by 157 or to only 53 TB cases from both countries combined. The impact of screening in Germany would probably prevent no cases in most years.

**Table 4:** Cases of TB Diagnosed among Foreign-Born Student-Visa Applicants.

Parameter	India	China	Germany
Cases diagnosed overseas	29.2	127.8	0
Cases in Class B-1 students during first year in U.S.^A^	17.9	34.7	0
Total cases by student’s first year in U.S.^B^	47.1	162.5	0

U.S. = United States; TB = Tuberculosis

A-Class B-1 indicates those who have an abnormal chest radiograph, signs and symptoms of TB, or known HIV infection during overseas screening;

B-All cases assumed to be imported into U.S. in absence of screening

The cost results differ by perspective. From the United States perspective, student screening programs would be cost saving if implemented in both India and China, but not Germany (Table 5). Total United States savings from implementing the program in both India and China were estimated at $2,693,106. Implementing the program in Germany would result in additional United States costs of just over $5,200, mainly for PHDs because of the increase in follow-up evaluations for persons who are Class B-1 upon arrival (Table 5).

**Table 5:** U.S. Costs Incurred with Two Programs for Treating TB in Foreign-Born Student-Visa Applicants.

Parameter	India		China		Germany	
	(N = 29,981)		(N = 58,015)		(N = 2,795)	
	No screening^A^	Overseas screening and treatment^B^	No screening^A^	Overseas screening and treatment^B^	No screening^C^	Overseas screening and treatment^B^
Student’s opportunity costs	$136,239	$58,468	$470,278	$121,557	$0	$237
Student’s out of pocket^D^	$269,164	$76,133	$929,118	$149,387	$0	$63
Insurance hospitalization^E^	$359,601	$96,607	$1,241,291	$186,674	$0	$0
PHD treatment	$246,125	$93,810	$849,589	$181,269	$0	$0
PHD follow-up in Class B-1 students^F^	NA	$216,587	NA	$587,598	NA	$4,668
DGMQ coordination of follow-up^G^	NA	$10,829	NA	$29,380	NA	$233
Total government costs^H^	$246,125	$321,226	$849,589	$798,247	$0	$4,901
Total U.S. costs	$1,011,129	$552,434	$3,490,276	$1,255,865	$0	$5,201
Difference in U.S. costs with overseas screening programs		Savings of $458,695		Savings of $2,234,411		Additional Cost of $5,201

DGMQ = Division of global migration and quarantine; PHD = Public health department; TB = Tuberculosis; U.S. = United States

A-All costs occur as a result of treating TB in the U.S. passively without active screening;

B-Includes domestic screening costs for those identified overseas as having elevated risk of developing TB as well as costs of treating TB detected through screening and TB detected passively;

C-No treatment costs incurred as no cases would be expected among German students in most years and in this scenario there would also be no additional screening required;

D-Includes travel expenses for follow-up and TB treatment in the U.S., 20% of hospitalization expenses and 70% of forfeited undergraduate tuition in hospitalized students;

E-Assumes student incurs 20% of hospitalization related expenses and insurance company incurs 80% of hospitalization expenses for TB;

F-Class B-1 indicates those who have an abnormal chest radiograph, signs and symptoms of TB, or known HIV infection during overseas screening and are encouraged to follow-up at U.S. PHD;

G-Assumes 5% of PHD follow-up costs in order to notify PHD of students arriving with elevated risk of TB and to document outcomes of medical follow-up;

H-Includes all PHD costs as well as DGMQ costs

From the overseas perspective, the program would have additional costs resulting from medical screening and opportunity costs; these would largely be borne by students and their families (Table 6). From the ‘combined’ societal perspective there would also be additional costs, primarily incurred overseas. For Chinese, German, and Indian students the overseas program was estimated to incur increased costs of $2,835,523, $502,285, and $439,845 respectively from the combined societal perspective (Table 7).

**Table 6:** Overseas Costs Incurred with Overseas TB Screening and Treatment Programs in Foreign-Born Student-Visa Applicants.

Parameter	India (N = 29,981)	China (N = 58,015)	Germany (N = 2,795)
	Overseas screening and treatment	Overseas screening and treatment	Overseas screening and treatment
Initial screening	$478,197	$3,187,344	$203,839
Sputum smears and cultures	$91,933	$339,052	$2,614
TB treatment^A^	$12,679	$54,018	$0
Student’s opportunity costs	$315,731	$1,489,520	$290,631
Total overseas costs	$898,540	$5,069,934	$497,084

TB = Tuberculosis;

A-Includes supervision of treatment by panel physician, medications, and hospitalization costs

**Table 7:** Cost per Case Averted from Being Imported into the U.S. when Comparing Two Programs for Treating TB in Foreign-Born Student-Visa Applicants.

Country	Program costs	Additional costs with overseas screening	Domestic cases 1^st^ year	Incremental # cases prevented from being imported	Incremental cost per case prevented from being imported^A^
No screening or treatment for Indian students^B^	$1,011,129	reference	47.1	reference	reference
Overseas screening and treatment for Indian studentst^C^	$1,450,974	$439,845	17.9	29.2	$15,063
No screening for Chinese students^B^	$3,490,276	reference	162.5	reference	reference
Overseas screening and treatment for Chinese students^C^	$6,325,799	$2,835,523	34.7	127.8	$22,187
No screening for German students^B^	$0	reference	0	reference	reference
Overseas screening and treatment for German students^C^	$502,285	$502,285	0	0	Not able to be calculated (division by zero)

TB = Tuberculosis; U.S. = United States

A- Derived by dividing the additional costs with overseas screening by the cases prevented from being imported into the United States;

B-Includes costs for treating imported TB cases in the U.S. with no overseas screening;

C-Includes overseas costs incurred while screening for and treating TB overseas, screening high risk students in the U.S., and treatment of active TB cases occurring in the U.S. after implementing overseas screening and treatment

We also estimated ICERs from the ‘combined’ perspective in order to compare the costs associated with the overseas screening program and no screening program with the changes in numbers of TB cases imported into the United States. The combined perspective ICER indicated that when both overseas and United States costs were included, each TB case averted from Chinese and Indian students would cost an additional $22,187 and $15,063, respectively (Table 7). We were not able to derive an ICER for Germany. This was because no TB cases were identified or prevented, so the denominator of the ratio (cost/outcome) was 0 and the ratio results were mathematically invalid.

Total United States, PHD, and student costs vary by wide ranges depending on the student’s country of origin and with overseas screening or no screening (Table 5). For example, if Indian students were screened overseas for TB, United States PHD expenditures were $310,397, while if there were no Indian students screened overseas for TB, United States PHD expenditures fell to $246,125 because students did not receive domestic TB screening until they presented with symptoms of disease. Also, overseas costs associated with implementing overseas screening programs ranged from a low of $497,084 for German students to $5,069,934 for Chinese students and $898,540 for Indian students (Table 6).

For the first sensitivity analysis, we assessed the change in proportion of suspected TB cases by plus and minus 50% on the ICER (the main outcome from the combined perspective that accounted for all international and domestic costs and benefits). When this parameter was changed by plus or minus 50%, the ICERs for China and India varied from $10,485 to $57,188 and $6,330 to $41,592, respectively (Table 8).

**Table 8:** One-Way Sensitivity Analyses on Cases Prevented,^A^ U.S.-Perspective Savings,^B^ and ICER^C^ associated with Implementing Overseas TB Screening in Foreign-Born Student-Visa Applicants.

Parameter	Outcome	India	China
Base Case	Cases Prevented^A^	29.2	127.8
	U.S. Savings^B^	$458,696	$2,234,411
	ICER^C^	$15,063	$22,187
Suspected TB up 50%^D^	Cases Prevented^A^	43.7	191.8
	U.S. Savings^B^	$688,046	$3,351,615
	ICER^C^	$6,330	$10,485
Suspected TBdown 50%^D^	Cases Prevented^A^	14.5	64.0
	U.S. Savings^B^	$229,349	$1,117,205
	ICER^C^	$41,592	$57,188
0.3% Class B-1 students diagnosed with TB in U.S.^E^	Cases Prevented^A^	29.2	127.8
	U.S. Savings^B^	$389,273	$2,107,871
	ICER^C^	$17,441	$23,159
3% of Class B-1 students diagnosed with TB in U.S.^E^	Cases Prevented^A^	29.2	127.8
	U.S. Savings^B^	$482,996	$2,357,273
	ICER^C^	$14,231	$21,209
Undergraduate students hospitalized forfeit 100% tuition	Cases Prevented^A^	29.2	127.8
	U.S. Savings^B^	$512,350	$2,449,564
	ICER^C^	$13,226	$20,504
Undergraduate students hospitalized forfeit 0% tuition	Cases Prevented^A^	29.2	127.8
	U.S. Savings^B^	$333,504	$1,732,386
	ICER^C^	$19,351	$26,115
Graduate students hospitalized forfeit 70% tuition	Cases Prevented^A^	29.2	127.8
	U.S. Savings^B^	$510,609	$2,442,585
	ICER^C^	$13,285	$20,558
Hospitalization costs up 50%	Cases Prevented^A^	29.2	127.8
	U.S. Savings^B^	$623,067	$2,893,546
	ICER^C^	$9,434	$17,030
Hospitalization costs down 50%	Cases Prevented^A^	29.2	127.8
	U.S. Savings^B^	$349,115	$1,794,987
	ICER^C^	$18,816	$25,626
Opportunity costs not included	Cases Prevented^A^	29.2	127.8
	U.S. Savings^B^	$380,925	$1,885,690
	ICER^C^	$6,914	$13,260

ICER = Incremental Cost-Effectiveness Ratio; TB = Tuberculosis; U.S. = United States;

A-Cases prevented denotes cases prevented from being imported into the U.S. by active overseas screening programs;

B-U.S. perspective savings or costs represents the total projected costs or savings in the U.S. with overseas screening programs;

C-The incremental cost-effectiveness ratio is from the combined perspective which combines both overseas and foreign costs;

D-Suspected TB includes those with abnormal chest radiograph, signs and symptoms of TB, and/or known HIV infection;

E-Class B-1 indicates those who have an abnormal chest radiograph, signs and symptoms of TB, or known HIV infection during overseas screening and are encouraged to follow-up at U.S. PHD;

In the second sensitivity analysis, we varied the change in the proportion of Class B-1 students diagnosed with TB after entering the United States from 0.3% to 3%. As a result of varying this parameter, the ICERs varied from $21,209 to $23,159 for China and $14,231 to $17,441 for India.

The third sensitivity analysis evaluated the effect of changing the amount of lost tuition for hospitalized students by using three scenarios: undergraduate students forfeit no tuition, undergraduate students forfeit all tuition, and graduate students forfeit 70% of tuition. When this parameter was changed, the ICERs for China and India varied from $20,504 to $26,115 and $13,226 to $19,351 respectively.

In the fourth sensitivity analysis we varied the hospital cost from 50% below the original value to 50% above the original value. In this sensitivity analysis, the ICER varied from $17,030 to $25,626 for China and $9,434 to $18,816 for India.

The final sensitivity analysis assessed the impact of opportunity costs. When opportunity costs were not included either overseas or in the United States, the ICER was $6,900 for India and $13,260 for China.

## Discussion

From the United States perspective, this new program would be cost savings and reduce the public health impact of TB on the domestic population. We demonstrated that implementing overseas screening and treatment programs in students arriving from China and India would result annually in 157 averted TB cases and savings of $2.7 million. A great deal of these savings would accrue to private insurance companies as their expenditures for TB-related hospitalizations would decrease yearly by more than $1.3 million (Table 5). For public health departments (PHDs) specifically, treatment costs with overseas screening would decline by approximately $821,000, and with increased screening costs of approximately $804,000 for follow-up in Class B-1 students, there would be a net savings of close to $17,000 (Table 5). From an overseas perspective increased costs would be incurred by students and students’ families from additional steps to apply for admission to United States universities and colleges. There is the possibility that implementation of medical exams and TB testing may encourage some students to enroll elsewhere. However other countries such as Australia, Canada, and France and others already require students to undergo screening for active TB [57].

Implementing overseas TB screening programs in students would be another step in a continuum of programs that have reduced the burden of TB among populations migrating to the United States. The most recent programmatic change was initiated in a staged approach beginning in 2007 with more stringent TB-culture based screening protocols as opposed to the previous TB-smear based screening protocols. Prior to the implementation of the TB-culture based screening, the number of imported TB cases among newly-arrived foreign-born persons was relatively constant over many years [58]. However, internal tracking of TB disease cases seems to indicate that as the TB-culture based screening has been implemented in more countries, the constant number of imported TB cases has been substantially reduced. For example, the number of cases in all foreign-born during their first year in the United States had been reduced from the constant 1,500 a year to about 925 as of 2011 [58]. Our data indicates that if the proposed screening had been implemented among students from high-volume, high-burden countries such as China, India, and Vietnam, at the same time as the culture based screening was initiated for immigrants and refugees, the number of TB cases imported by the first year foreign-born could have been reduced even further to about 725 in 2011 (See S3 Table).

Our results probably understated the true impact of how overseas screening programs in these countries would benefit domestic PHDs and insurers because our model did not include multidrug-resistant TB (MDR-TB) or TB transmission once a student with active disease enters the United States. Even though MDR-TB is extremely expensive and time consuming to treat, the prevalence is low, so including MDR-TB in our model would not have substantively changed the overall results. As an example, the TB Indicator Data showed that 1.8% of Chinese immigrants diagnosed with TB had MDR-TB. Accordingly, without overseas screening, three of the 163 cases imported in Chinese students would have been MDR-TB cases. This would have changed the domestic costs associated with TB treatment in these students from $3.5 million to $3.6 million (See S1 Appendix).

Conversely, our analysis indicated that TB screening in German students would lead to increased costs, even from the United States perspective. This was largely because of the increased number of persons who were Class B-1 needing PHD follow-up, and the fact that our model estimated no cases in these students. Our findings were in line with those of other publications suggesting that screening for TB in otherwise healthy persons from low-incidence countries is low yield [59–61].

United States universities and colleges could lose anticipated tuition from foreign-born students diagnosed with TB through overseas screening and treatment programs as students cancel or delay enrollment while finishing treatment. However, some schools may have a wait list to replace unavailable students. The ability of schools to replace students depends on several factors that we could not account for in modeling, such as the timing of overseas examinations, how soon foreign-born students receive acceptance at United States colleges, and school policies on wait lists. Universities, especially those receiving a large volume of foreign-born students, may have to adjust their policies and procedures so that any economic damages from implementation of overseas TB screening would be minimized.

In our analysis, we assumed that treatment of cases diagnosed after arrival would take place at PHDs, in part because medical clinics or health professionals diagnosing TB are strongly encouraged to notify state and local health departments [62]. In some instances students may be treated in university clinics. To the extent that students are treated at university clinics instead of PHDs, the treatment costs allocated to PHDs would be shifted to university clinics. However, this shift would be unlikely to have an appreciable effect on the total costs from the United States perspective or the ICERs, which are calculated from a combined perspective.

The use of chest radiographs exposes the students to radiation that is associated with an increased risk of developing cancer. However, most students will receive only one chest radiograph with a small additional risk. Estimates indicate that approximately one case of radiation induced cancer occurs for every million chest radiographs conducted [63]. The authors do acknowledge that in populations with low TB prevalence, the risks of TB screening with chest radiographs may offer little additional public health benefit and might incur unnecessary or unjustified costs from a medical perspective.

We valued students’ opportunity costs because they would be heavily impacted by implementation of an active overseas screening program. Occupational wage data are often used to value opportunity costs [34], but doing so in students would be inappropriate because foreign student visas restrict their opportunities for paid work. We used hourly country-specific per capita GDP to reflect student economic activity with regards to consumption (e.g., food, housing, clothes) that constitutes the majority of total GDP in most countries [64,65]. Our method of valuing the opportunity costs of foreign-born students in the United States was conservative at $5.49 hourly and less than the minimum wage. Further, without including opportunity costs, the results of implementing overseas screening programs would appear to be more cost-effective than the results of our base-case analysis indicate (Table 7) and we did not want to slant the results by not including the impact on students.

Our benefit calculations are understated because we were unable to quantitatively estimate the benefits foreign-born students derive from obtaining an American education. Qualitatively, though foreign-born students who are interviewed clearly believe that there is a high prestige associated with degrees from an American institution [66] as there is worldwide recognition of the excellence of United States colleges and universities [67]. Many students also cite the advanced technology and hands on training available at United States universities and colleges [68]. In addition, foreign-born students value the flexibility at United States colleges in terms of the wide variety of courses offered and freedom to decide on a major concentration after taking their initial courses [68]. United States universities and colleges also tend to provide more professional connections within a student’s field of study [67].

We were unable to determine how the prevalence of TB would differ in students as opposed to the overall immigrant population because TB Indicator Data are reported for general immigrant populations of all ages. In general, younger adults have a lower prevalence of TB than older adults, but the magnitude of the difference varies by country [69–74]. We account for the potential discrepancy in population-wide TB rates and young adult TB rates in the sensitivity analysis, where the proportion of students with suspected TB is reduced to half of that in the actual data which would result in the ICERs being higher than those that were presented in our base case scenario.

Because of the lack of TB Indicator Data for Germany, we assumed German students were represented by an average of the Canadian and French TB rates, and acknowledge that direct German data would have been better. However, at the time of analysis, Germany had not yet implemented the most recent TB TIs, which require TB test result reports by panel physicians to TB Indicator Data. Further, we had no other data that were uniform across all other low-incidence countries. For example, we had panel physician charge data for Germany but not for France.

In spite of limitations, the TB Indicator Data represented the best available data, allowing us to directly estimate the proportion of students that would need to receive sputum smears and cultures and estimate numbers of TB cases diagnosed in a way that other data sets would not.

While (LTBI) is considered by PHDs to be an important contributing factor to the numbers of TB cases diagnosed and/or imported into the United States LTBI inclusion was beyond the scope of the current project. However, we intend to conduct analyses in the future which examine the cost and benefits of LTBI screening and treatment of class B-1 students once they arrive in the United States

## Conclusions

Expansion of the current CDC TB screening and treatment program already in place for United States bound refugees and immigrants to include United States bound students would help reduce the TB disease burden in the United States. From the United States perspective, it would be cost saving to implement overseas screening programs in countries with relatively high rates of TB, such as China and India, but it would not be cost saving to implement active screening in countries such as Germany, where there are only limited cases of TB. Implementation of screening programs in students from high-incidence countries could potentially save the United States health system millions of dollars annually, however there would be increased costs incurred overseas where students are screened.

## Supporting Information

S1 Appendix Supplementary Information. (DOCX)

S1 Table Summary of Tuberculosis Screening Results in 2012. (DOCX)

S2 Table Proportion of B1 Immigrants Diagnosed with TB at Follow-up in U.S. Health Departments. (DOCX)

S3 Table Potential TB Cases Diagnosed Overseas in Foreign-Born Student-Visa Applicants. (DOCX)
