# Supplementary material for: Cost-Effectiveness of Screening and Treating Foreign-Born Students for Tuberculosis before Entering the United States
Source: PLoS One. 2015 Apr 29;10(4):e0124116. doi: 10.1371/journal.pone.0124116 (PMC4414530; doi:10.1371/journal.pone.0124116)
Supplement: S1 Appendix — (DOCX) [file pone.0124116.s001.docx]

**S1 Appendix. Supplementary Information**

The ICER formula used in this model looked at the cost of TB cases averted, and therefore used this type of ICER formula: (Cost1-Cost2)/(Outcome2-Outcome1). For this study specifically, the formula used was ([(cost of overseas screening) – (cost of no overseas screening)]/ [(TB cases diagnosed in United States with no overseas screening) – (TB cases diagnosed in United States with overseas screening)]). Had the ICER formula looked at, for example, quality adjusted life years gained, the formula would have been (Cost 1-Cost 2)/(Outcome 1-Outcome 2); or in other words, in the denominator the Outcomes would have been the reverse of the formula we used in our model. See, for example, <http://www.cdc.gov/owcd/eet/costeffect2/fixed/4.html> .

**Students:** During the 2011/12 academic year there were nearly 765,000 foreign-born students enrolled in US degree and non-degree programs [1]. Our hypothetical study cohort included degree and non-degree students, but confined cost calculations to undergraduate student tuition rates.

**Screening process definitions and explanations:**

Panel physicians: licensed physicians in the country of origin who are extended an agreement by the Consular Sections of United States. Embassies to screen immigrants and refugees.

The panel physician process is already established to screen immigrants (primarily) seeking entry to the United States, so screening students might require supplementing the current systems with more space and personnel. However, issues associated with expanding capacity were beyond the scope of this study and not included in our model.

Technical Instructions: DGMQ develops Technical Instructions for the medical exams administered by panel physicians. These include instructions for testing and treating TB (TB TI). The most recently implemented version is the Culture and Directly Observed Therapy Technical Instructions (CDOT TB TI) [2]. Panel physicians apply the TB TI as part of the overall medical examinations, and those countries operating under the CDOT TB TI also supply DGMQ with documentation of exam results. The results of screening and treatment are maintained in the TB Indicator Data. In the model of the overseas screening strategy, all students would receive chest radiographs and clinical examinations to test for TB [2]. Students were classified as having suspected TB if they had an abnormal finding on chest radiography, signs and symptoms of TB, or known HIV infection. Students were affirmatively diagnosed as having active TB that needed treatment either through positive sputum smear and culture tests or clinical diagnosis.

TB medical visa classifications:

- No TB classification indicating no positive test results
  - In the most current screening and treatment process for adults over age 14 all applicants receive a chest radiograph as initial screening [2,3]. Persons with normal radiographic findings, no signs or symptoms of TB, and no self-reported HIV infection are medically cleared without further testing.
- Class A indicates active TB; the person cannot travel to the United States until successfully treated and retested as cleared of disease and they are then medically reclassified as B1.
  - Applicants who are diagnosed with TB and are given Class A medical designations on their visas must receive Directly Observed Therapy (DOT) supervised by a panel physician for their TB.
  - After successfully completing treatment and receiving confirmation of cure through negative post-treatment sputum smears and cultures, the applicant receives a Class B-1 visa designation and can reapply to enter the United States.
- Class B-1 medical designations on visas should receive medical follow up once the person has arrived in the United States. B1 indicates:
  - the possibility but not proof of active TB;
  - previous active TB that has been successfully treated and cleared;
  - Applicants with abnormal radiographic findings that could indicate TB, signs and symptoms of TB, or self-reported HIV infection are required to have three sputum smear and culture tests. These applicants are suspected of having TB. If the results of all sputum smears and cultures are negative, a person is medically cleared with a Class B1 visa designation.

**Estimating the total number of student TB cases imported:**

In our model, we grouped all of the TB cases into two broad categories: those cases that would have been detected at the time of overseas screening (prevalent cases) and those cases which would have been detected by the end of the student’s first year in the United States but after overseas screening (incident cases). Accordingly, the total number of TB cases in students was estimated by the following: Total TB cases = prevalent cases detectable at time of overseas screening + incident cases detectable after overseas screening. Under the current situation where there are no overseas screening programs prevalent cases are imported into the United States. However, with overseas screening programs, prevalent cases would be treated overseas so that they would not be imported into the United States. Incident cases would be imported into the United States regardless of whether overseas screening was conducted or not. Therefore under the current scenario of no overseas screening, the number of TB cases imported into the U.S. would equal the sum of the prevalent and incident cases, and all of these cases would be detected passively in the United States. If overseas screening programs were to be implemented only incident cases would be imported into the United States.

**Estimating the number of prevalent TB cases:**

The number of prevalent cases was estimated with the TBI Indicator Data. During 2012, the prevalence of active TB in the TB Indicator Data was 220 cases per 100,000 for persons screened in China and 99 cases per 100,000 for persons screened in India. No TB Indicator Data were available for Germany at the time of analysis because they had not yet implemented the most recent TB TIs for screening and treatment of TB in United States bound immigrants and therefore were not reporting TB test results to the data set, so we used available data from the low incidence countries of Canada and France. We assumed that the number of prevalent TB cases was proportional to the number of students being screened. Because China had an estimated 58,015 new students, we multiplied 0.58 (58,015/100,000) by 220 in order to estimate that there were approximately 128 prevalent cases at the time of screening.

**Estimating the incident number of TB cases:**

The incident cases were assumed to occur in students that would have had a B-1 classification if overseas screening had occurred, and all incident cases were assumed to be treated after arrival in the United States. As described in the screening process definitions and explanations above, Class B-1 individuals include those with abnormal radiographic findings, signs or symptoms of TB or known HIV infection. Upon United States arrival, Class B-1 immigrants are encouraged to follow-up at receiving health departments for medical care and screening. Over three quarters of immigrants present to this medical follow-up [4]. The outcomes of these follow up exams are recorded in data maintained by the CDC (electronic disease notification system). At the time of the analysis, the data in the electronic disease notification system indicated that 2.30% of Indian immigrants with a Class B-1 classification and 1.67% of Chinese immigrants with a Class B-1 classification were diagnosed with TB at the follow-up examinations. We assumed that if students were screened overseas, they would have the same country specific proportion of Class B-1 individuals diagnosed with TB after arrival in the United States. The incident cases could be detected at follow-up (active detection) or they could be diagnosed passively in those students who did not present to follow up.

**DOT treatment practices at the PHD level:**

Discussions with CDC TB experts and PHD personnel indicated that in some cases PHDs took DOT treatment to the patient, while in other, the patient traveled to the PHD.

**Potential impact of screening students from multiple countries**

We also used TB Indicator Data and Data on the number of students coming to the United States to estimate the impact of a potential program to screen students for TB from multiple countries. Nearly 765,000 foreign-born students resided in the United States during the 2011/12 academic year and about 76% of these originated from 39 countries that had submitted TB Indicator data at the time of the analysis [1]. The TB Indicator Database contains aggregate results for the screening of refugees residing in camps in addition to immigrants. Because refugees as a group often have higher rates of TB than general populations, we excluded students from countries with refugee camps, including Nepal, Thailand, Malaysia, Kenya, Ethiopia, Tanzania, Uganda, and Jordan. There were 548,040 foreign-born students in the United States from the remaining countries without refugee camps, or approximately 72% of all foreign-born students.

Approximately 30% of foreign-born students are newly enrolled, so we assumed that this same proportion, or 163,864 students, would be in their first school year [5]. We applied the student originating country-specific TB case rates from the TB Indicator Data to this first-year student population. For example, we estimated that there would have been 4,656 new Vietnamese students. TB Indicator Data documented 893 cases per 100,000 of Vietnamese immigrants screened. Therefore we estimated that 42 TB cases might be prevented in Vietnamese students in their first school year in the United States by overseas TB screening and treatment.

**Impact of including multidrug resistant TB**

In our analysis, we estimated that without overseas screening, 162.5 cases of drug susceptible TB were expected to be imported into the United States by Chinese students. These 162.5 cases were associated with $3,490,276 in costs or an average of $21,479 per case (2011 United States Dollars). The TB Indicator Data shows that out of all immigrants diagnosed with TB, 1.8% of them had multidrug-resistant TB (MDR TB). Accordingly, if in a given year there were 163 cases of TB imported into the U.S. by Chinese students, three of the cases would be MDR TB.

Holland and colleagues estimated that an MDR TB case in the U.S. was associated with $51,220 in costs in 2009 United States Dollars or $54,581 in 2011 United States dollars [6-7]. Thus, the costs associated with three MDR-TB cases in 2011 United States Dollars would be $163,743. However, in this analysis, we also include the cost of forfeited tuition. The Holland analysis assumes a hospitalization rate of 70% [6]. If two of the Chinese students in our cohort with MDR-TB were hospitalized and had to forfeit 70% of their tuition, there would be $14,838 worth of forfeited tuition [8]. Accordingly, the total cost for treating the MDR cases in the United States would be estimated at $178,581. The cost for treating 160 cases of drug susceptible TB would be $3,436,160. Hence the total cost for treating both drug susceptible and MDR cases in Chinese students would be approximately $3.6 million.

**References**

1. Institute of International Education. International student totals by place of origin, 2010/11 - 2011/12. Available: <http://www.iie.org/Research-and-Publications/Open-Doors/Data/International-Students/All-Places-of-Origin/2010-12>

2. Centers for Disease Control and Prevention. Tuberculosis screening and treatment technical instructions (TB TIs) using cultures and directly observed therapy (DOT) for panel physicians. Available: <http://www.cdc.gov/immigrantrefugeehealth/exams/ti/panel/tuberculosis-panel-technical-instructions.html>.

3. Lowenthal P, Westenhouse J, Moore M, Posey DL, Watt JP, et al. Reduced importation of tuberculosis after the implementation of an enhanced pre-immigration screening protocol. Int J Tuberc Lung Dis. 2011;15: 761-766.

4. Liu Y, Weinberg MS, Ortega LS, Painter JA, Maloney SA. Overseas screening for tuberculosis in U.S.-bound immigrants and refugees. N Engl J Med. 2009;360: 2406-2415.

5. Institute of International Education. New international student enrollment, 2004/05 - 2011/12. Available: <http://www.iie.org/Research-and-Publications/Open-Doors/Data/International-Students/New-International-Enrollment/2004-12>.

6.Holland DP, Sanders GD, Hamilton CD, Stout JE. Strategies for treating latent multiple-drug resistant tuberculosis: a decision analysis. PLoS One. 2012;7:e30194.

7.Bureau of Labor Statistics. Consumer Price Index-all urban consumers, medical care. Available: http://data.bls.gov/timeseries/CUUR0000SAM?output_view=pct_12mths.

8. National Center for Education Statistics. Average undergraduate tuition and fees and room and board rates charged for full-time students in degree-granting institutions, by control and level of institution and state or jurisdiction: 2009 - 10 and 2010 - 11. 2011. Available: http://nces.ed.gov/programs/digest/d11/tables/dt11_350.asp.
