# Supplementary material for: Cost-Effectiveness of Screening and Treating Foreign-Born Students for Tuberculosis before Entering the United States
Source: PLoS One. 2015 Apr 29;10(4):e0124116. doi: 10.1371/journal.pone.0124116 (PMC4414530; doi:10.1371/journal.pone.0124116)
Supplement: S1 Table — (DOCX) [file pone.0124116.s002.docx]

**S1 Table. Summary of Tuberculosis Screening Results in 2012^A^**

| **Country** | **Number of applicants screened^B^** | **# of applicants with suspected TB^C^** | **% of applicants with suspected TB** | **# of applicants diagnosed with TB** | **Calculated TB incidence rate per 100,000 at panel site** | **TB Incidence Rate Per 100,000 (WHO)** |
| --- | --- | --- | --- | --- | --- | --- |
| Argentina | 372 | 8 | 2.2 | 0 | 0 | 26 |
| Canada | 2146 | 14 | 0.7 | 0 | 0 | 4.5 |
| China | 51397 | 1948 | 3.8 | 113 | 220 | 75 |
| Colombia | 9262 | 172 | 1.9 | 4 | 0 | 34 |
| Costa Rica | 375 | 15 | 4.0 | 0 | 0 | 12 |
| Egypt | 1342 | 8 | 0.6 | 0 | 0 | 17 |
| France | 487 | 2 | 0.4 | 0 | 0 | 4.3 |
| India | 20160 | 548 | 2.7 | 20 | 99 | 181 |
| Japan | 1224 | 8 | 0.7 | 0 | 0 | 20 |
| Jordan | 8877 | 34 | 0.4 | 0 | 0 | 6 |
| Mexico | 92000 | 1975 | 2.1 | 44 | 48 | 23 |
| Philippines | 38158 | 5025 | 13.2 | 396 | 1038 | 270 |
| Singapore | 491 | 1 | 0.2 | 1 | 204 | 37 |
| South Korea | 5784 | 202 | 3.5 | 2 | 34 | 100 |
| United Kingdom | 5371 | 43 | 0.8 | 0 | 0 | 14 |
| Vietnam | 24978 | 2317 | 9.3 | 226 | 893 | 199 |

TB=Tuberculosis

A-Data based on applicants applying for permanent residence to the United States in countries that were using the Culture and Directly Observed Therapy Technical Instructions.

B-Number of applicants comes from the panel sites in the country submitting data to the CDC on screening results.

C-Applicants were classified as having suspected TB if they had an abnormal chest ray, a medical history which might be indicative of TB, or known HIV infection.

**S2 Table. Proportion of B1 Immigrants^A^ Diagnosed with TB at Follow-up in U.S. Health Departments**
