# Supplementary material for: Cost-Effectiveness of Screening and Treating Foreign-Born Students for Tuberculosis before Entering the United States
Source: PLoS One. 2015 Apr 29;10(4):e0124116. doi: 10.1371/journal.pone.0124116 (PMC4414530; doi:10.1371/journal.pone.0124116)
Supplement: S2 Table — (DOCX) [file pone.0124116.s003.docx]

**S2 Table. Proportion of B1 Immigrants^A^ Diagnosed with TB at Follow-up in U.S. Health Departments**

| **Country** | **Proportion diagnosed with tuberculosis at follow-up** |
| --- | --- |
| China | 1.67% |
| Germany | 0% |
| India | 2.33% |

TB=Tuberculosis

A-B1 Immigrants includes those were treated for active tuberculosis overseas and subsequently verified to be cured by a panel physician or those that were classified as having suspected tuberculosis during overseas exams as a result of an abnormal chest ray, a medical history which might be indicative of TB, or known HIV infection
