# Supplementary material for: Cost-Effectiveness of Screening and Treating Foreign-Born Students for Tuberculosis before Entering the United States
Source: PLoS One. 2015 Apr 29;10(4):e0124116. doi: 10.1371/journal.pone.0124116 (PMC4414530; doi:10.1371/journal.pone.0124116)
Supplement: S3 Table — (DOCX) [file pone.0124116.s004.docx]

**S3 Table. Potential TB Cases Diagnosed Overseas in Foreign-Born Student-Visa Applicants**

| Country | Estimated number of new students | Rate of TB diagnosed in overseas screening for 2012^A^ | Potential TB cases detected overseas^B^ |
| --- | --- | --- | --- |
| China | 58,015 | 220 | 128 |
| Vietnam | 4,656 | 893 | 42 |
| India | 29,981 | 99 | 30 |
| Philippines | 955 | 1,038 | 10 |
| South Korea | 21,616 | 34 | 7 |
| Singapore | 1,347 | 204 | 3 |
| Mexico | 4,154 | 48 | 2 |
| All other countries in data | 43,140 | 19 | 8 |

TB=Tuberculosis

A-Rate given as number of cases per 100,000 population and is taken from TB Indicator Data which records the TB related screening outcomes for populations migrating to the U.S.; B-Rounded to nearest whole number
